# Hypermethylated gene ANKDD1A is a candidate tumor suppressor that interacts with FIH1 and decreases HIF1α stability to inhibit cell autophagy in the glioblastoma multiforme hypoxia microenvironment

**DOI:** 10.1038/s41388-018-0423-9

**Published:** 2018-08-06

**Authors:** Jianbo Feng, Yan Zhang, Xiaoling She, Yingnan Sun, Li Fan, Xing Ren, Haijuan Fu, Changhong Liu, Peiyao Li, Chunhua Zhao, Qiang Liu, Qing Liu, Guiyuan Li, Minghua Wu

**Affiliations:** 1 Hunan Provincial Tumor Hospital and the Affiliated Tumor Hospital of Xiangya Medical School, Central South University, Changsha, 410013 Hunan China; 2 The Key Laboratory of Carcinogenesis of the Chinese Ministry of Health, The Key Laboratory of Carcinogenesis and Cancer Invasion of the Chinese Ministry of Education, Cancer Research Institute, Central South University, Changsha, 410008 Hunan China; 3 The Second Xiangya Hospital, Central South University, Changsha, 410011 Hunan China; 4 Department of Biochemistry, University of California, Riverside, CA 92521 USA; 5 The Third Xiangya Hospital, Central South University, Changsha, 410011 Hunan China; 6 The Xiangya Hospital, Central South University, Changsha, 410008 Hunan China

**Keywords:** CNS cancer, Autophagy

## Abstract

Ectopic epigenetic mechanisms play important roles in facilitating tumorigenesis. Here, we first demonstrated that ANKDD1A is a functional tumor suppressor gene, especially in the hypoxia microenvironment. ANKDD1A directly interacts with FIH1 and inhibits the transcriptional activity of HIF1α by upregulating FIH1. In addition, ANKDD1A decreases the half-life of HIF1α by upregulating FIH1, decreases glucose uptake and lactate production, inhibits glioblastoma multiforme (GBM) autophagy, and induces apoptosis in GBM cells under hypoxia. Moreover, ANKDD1A is highly frequently methylated in GBM. The tumor-specific methylation of ANKDD1A indicates that it could be used as a potential epigenetic biomarker as well as a possible therapeutic target.

## Introduction

The classic hallmark of human cancer genomes is aberrant DNA methylation, including the genome-wide DNA hypomethylation and hypermethylation of CpG island-associated gene promoters. The latter leads to the epigenetic silencing of tumor suppressor genes, thereby facilitating the initiation and progression of cancer [1]. DNA methylation alterations have been widely reported in human glioma [2], one of the major central nervous system diseases that ranks in the top place in the incidence of primary intracranial tumors and level of malignancy.

Several genes were found to be repressed by promoter-associated CpG island hypermethylation in human GBM and other glioma subtypes [3]. For example, hypermethylation of the MGMT promoter-associated CpG island has been shown in a large percentage of GBM patients, and patients with MGMT hypermethylation showed sensitivity to alkylating agents such as temozolomide [4]. Interestingly, hypermethylation can also be genetically encoded; mutations in some genes correlated positively with hypermethylation (e.g., IDH1, TET, and BRAF), indicating the existence of a complex glioma CpG island methylation phenotype (gCIMP) [5, 6]. Thus, there is currently great interest in characterizing aberrant DNA methylation in human glioma tumors to identify aberrantly functioning molecular pathways and tumor subtypes. Moreover, the hypermethylation of aberrant tumor suppressor genes is not only a mechanism about tumor initiation, but also a biomarker for tumor diagnosis and prognosis prediction [7, 8].

Hypoxia conditions are caused in many solid tumors (including high-grade glioma) by abnormal structure and function of the micro-vessels. Tumor hypoxia has been often associated with resistance to cancer treatment, increased risk of invasion and metastasis, and poor prognosis [9]. Because hypoxia-inducible factor 1α (HIF1α) is the major regulator of tissue oxygen homeostasis and HIF1α expression closely correlates with tumor growth and invasion [10], HIF1α is considered to be responsible for hypoxia-mediated cancer progression.

In previous studies, we adopted Methyl–DNA immunoprecipitation (MeDIP) and NimblegenCpG promoter microarrays to identify differential DNA methylation sequences between primary glioma and normal brain tissue samples. We have previously identified nine new hypermethylated genes and six new hypomethylated genes in glioma [11]. We have reported the functions of some genes in glioma, including LRRC4 [12–14], CPEB1 [15], LMO3 [16], and PRDM16 [17].

Here, we reported a new hypermethylated gene, ANKDD1A (ankyrin repeat and death domain-containing 1A), which acts as a tumor suppressor in GBM, especially under hypoxia. ANKDD1A is located at 15q22.31 and contains nine ankyrin repeats and one death domain. The ankyrin repeat is one of the most common protein–protein interaction motifs in nature, and the repeat has been found in proteins of diverse functions, such as transcriptional activator, transporters, inflammatory responses, and signal transducers [18–21]. We confirmed for the first time that ANKDD1A directly interacted with the hypoxia-inducible factor 1 alpha subunit inhibitor (HIF1AN, also known as FIH1), which hydroxylates the Asn803 residue in the C-terminal-activating domain (C-TAD) of HIF1α and inhibits the transactivation function of HIF1α [22]. In addition, FIH1 binds to von Hippel–Lindau (VHL) tumor suppressor protein, which also functions as a transcriptional corepressor inhibiting HIF-1α transactivation [23]. Our findings revealed that the regain of ANKDD1A expression resulted in reduced transactivation function and stability of HIF1α, which suppressed GBM cells from adapting to hypoxia, inhibited cell autophagy and induced apoptosis in hypoxic microenvironment.

## Results

### Aberrant promoter hypermethylation conferred decreased expression of ANKDD1A in glioma

**Fig. 1:**
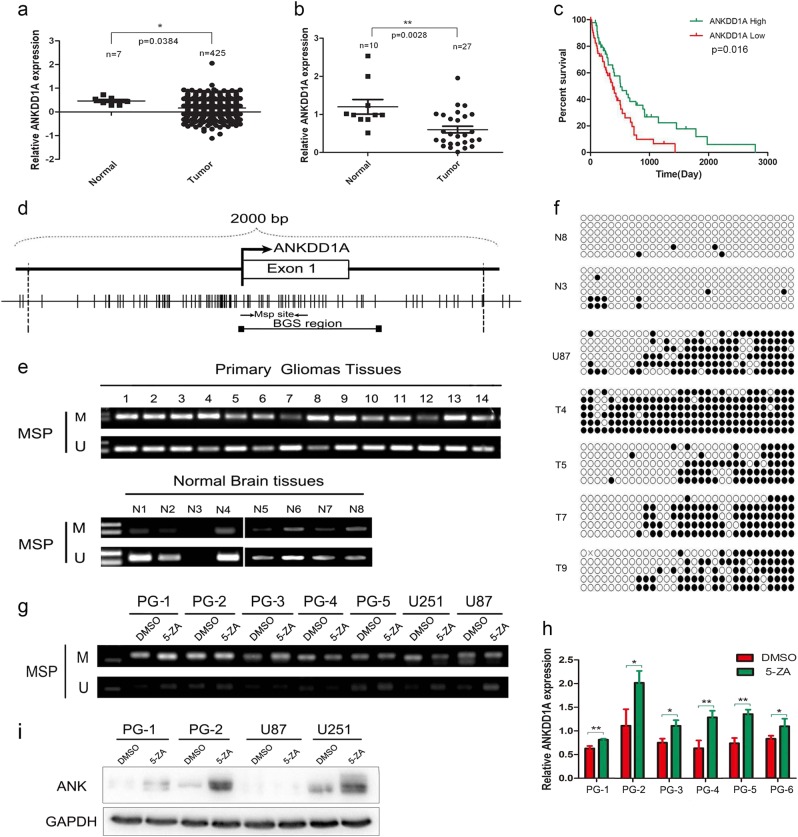
Hypermethylation of ANKDD1A in glioma and restoration of ANKDD1A expression by demethylation treatment. **a** Analysis of ANKDD1A expression in TCGA glioma gene expression data. The expression of ANKDD1A is much lower in glioma than in normal brain tissue. *P < 0.05. **b** Real-time qPCR was used to detect the expression of ANKDD1A in glioma. The expression of ANKDD1A is much lower in glioma than in normal brain tissues. **P < 0.01. **c** The correlation between ANKDD1A expression in glioma and the OS of the glioma patients. The glioma patients with higher ANKDD1A expression had a favorable survival time. The Kaplan–Meier method was used for this analysis. **d** Schematic structure of the CpG island of ANKDD1A, exons, methylation-specific PCR primer sites, and bisulfite genomic sequencing region. Each short vertical line in the bottom panel is one CpG site. **e** Methylation status of the ANKDD1A promoter in primary glioma tissues was analyzed by methylation-specific PCR. N normal tissue; Primary glioma tissues: samples 1–10 are GBM, samples 11–12 are grade II astrocytoma, samples 13–14 are grade III astrocytoma. M methylated; U unmethylated. **f** Methylation was analyzed by bisulfite genomic sequencing analysis of 33 CpG sites within the ANKDD1A promoter CpG island. N normal tissue, T tumor tissue. T4, T5, T7, and T9 are GBM samples. Each circle is one CpG site, and filled circles are methylated CpG sites. **g**–**i** Demethylation of ANKDD1A by 5-aza-2-deoxycytidine-induced ANKDD1A re-expression at the mRNA and protein levels, and this demethylation was confirmed by methylation-specific PCR. PG cells are primary cells derived from glioma patient. *P < 0.05, **P < 0.01

The TCGA database analysis (G4502A) indicated that ANKDD1A had decreased the expression in glioma compared with normal brain tissues (Fig. 1a), and this expression pattern was further confirmed by real-time PCR in glioma tissues (*n* = 27) and normal brain tissues (*n* = 10) (Fig. 1b). Most tumor samples had low ANKDD1A expression levels (17/27), and others had relatively medium or high expression levels (10/27). However, almost all of the normal brain tissues had medium or high ANKDD1A expression levels (9/10), but only 1/10 had a low ANKDD1A expression level. Kaplan–Meier analysis also demonstrated that there were longer survival times for glioma patients with relatively high ANKDD1A than for those with lower ANKDD1A expression. Thus, high ANKDD1A levels act as a favorite prognosis factor for glioma patients (Fig. 1c). The methylation of DNA is generally regarded as one of the most important epigenetic modifications that lead to gene silencing. By MethPrimer analysis, we found that the ANKDD1A promoter contained a CpG island (region −400 bp to +400 bp from transcription start site; Fig. 1d), indicating that hypermethylation may cause the decreased expression of ANKDD1A in glioma. Thus, we further designed bisulfite sequencing PCR (BSP) and methylation-specific PCR (MSP) to validate the hypermethylation of ANKDD1A in glioma. Methylation-specific PCR showed that ANKDD1A was hypermethylated in most glioma tissues (12/14) but only partially methylated in normal brain tissues (1/8) (Fig. 1e). Similarly, using high-resolution bisulfite genomic sequencing to analyze the methylation state in 33 CpG sites within the CpG island of the ANKDD1A gene, we found that there were more (20/33) methylated CpG sites in the ANKDD1A gene promoter in glioma compared to normal brain tissues (Fig. 1f). Next, we investigated whether pharmacologic demethylation restored ANKDD1A expression. Glioma cells were treated with the DNA methyltransferase inhibitor 5-aza-2-deoxycytidine. The treatment increased unmethylated alleles (Fig. 1g), accompanied by an increase in ANKDD1A mRNA and protein expression (Fig. 1h, i). Overall, these results demonstrated that CpG hypermethylation at the ANKDD1A promoter region directly contributed to the decreased expression of ANKDD1A in glioma.

### Ectopic expression of ANKDD1A reduced the proliferation and invasion of astrocytoma cells

**Fig. 2:**
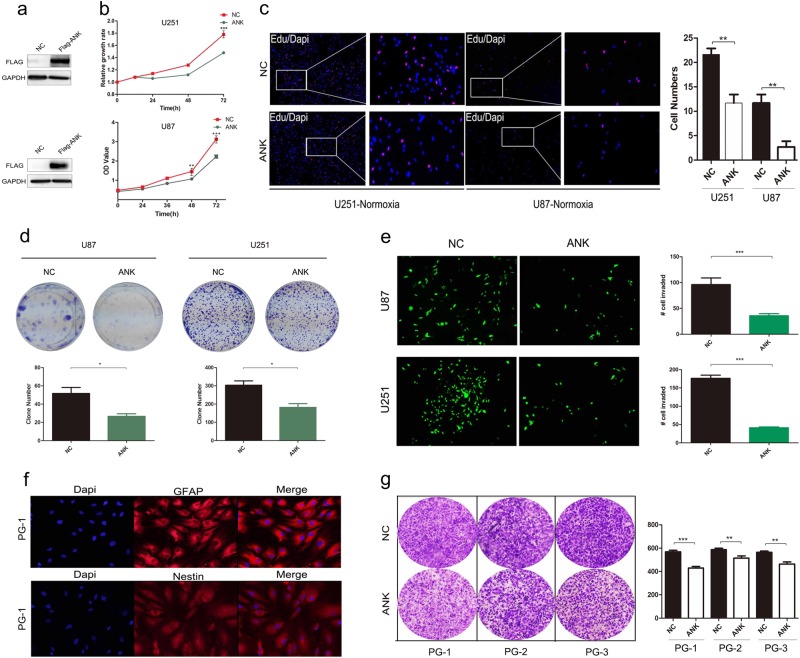
Re-expression of ANKDD1A inhibited GBM cell proliferation and invasion under normoxia. **a** The successful overexpression of ANKDD1A-Flag protein in GBM U251 (up)/U87 (down) cells was detected by immunoblotting. **b** The effect of ectopic ANKDD1A expression on GBM U251 and U87 cell proliferation was assessed by the CCK-8 cell growth assay. ANK cells transfected with Flag-ANKDD1A plasmid, NC cells transfected with Flag-NC plasmid, **P < 0.01, ***P < 0.001. **c** Expression of ANKDD1A inhibited DNA replication in GBM cells, as determined by EDU staining. The picture on the right is the enlargement of the white box in the left pictures. **P < 0.01. **d** Ectopic expression of ANKDD1A inhibited U251 and U87 cell clone formation ability, as analyzed by colony formation assay and crystal violet staining after 14 days; clone numbers were quantified. *P < 0.05. **e** U251 and U87 cells were stably transduced with lenti-vector-GFP or lenti-ANK-GFP; transwell assay was used to detect the cell invasion ability. ***P < 0.001. **f** The patient-derived primary cells were identified by immunofluorescence staining with GFAP and Nestin antibody. PG-1 represents primary cells that were derived from high-grade glioma patients. **g** Transwell assay was used to detect the invasion inhibitory effect of re-expression of ANKDD1A in patient-derived primary cells. PG-1, PG-2, and PG-3 are primary cells derived from glioma patients. *P < 0.05, **P < 0.01, ***P < 0.001

Ectopic expression of ANKDD1A (Fig. 2a) in U251 and U87 cells suppressed GBM cell proliferation (Fig. 2b). Moreover, inhibition of GBM cell proliferation by ANKDD1A was also confirmed by EDU staining assay (Fig. 2c). The number of EDU-positive cells significantly decreased upon ANKDD1A transfection (ANK group) compared to the NC group, suggesting that ANKDD1A observably inhibited the growth of GBM cells. To test the tumor suppressive function of ANKDD1A, we analyzed the growth characteristics and invasion ability (the upper chambers containing Matrigel coating mean that this assay measures an invasion phenotype but not migration phenotype of cells) in cells stably transduced with lenti-NC-GFP or lenti-ANK-GFP (Fig. 2d, e). We found that the colonies formed by the ANK-transfected cells were fewer and smaller than those formed by the vector-transfected cells, and the invaded cell numbers were also decreased compared to the vector-transfected cells. Moreover, to examine the clinical effect of ANKDD1A-inhibiting glioma proliferation in more detail, we isolated and cultured primary cells derived from glioma patients (hereafter referred to as PG). These cells were identified by immunofluorescence staining with GFAP and Nestin antibody (Fig. 2f), two known glioma molecular makers. As shown in Fig. 2g, the re-expression of ANKDD1A in primary cells reduced cell invasion in a Matrigel invasion assay (Fig. 2g). Together, these data provided direct evidence showing that ANKDD1A inhibited GBM cell proliferation and invasion.

### ANKDD1A bound to FIH1 by the conserved ankyrin repeat domain

**Fig. 3:**
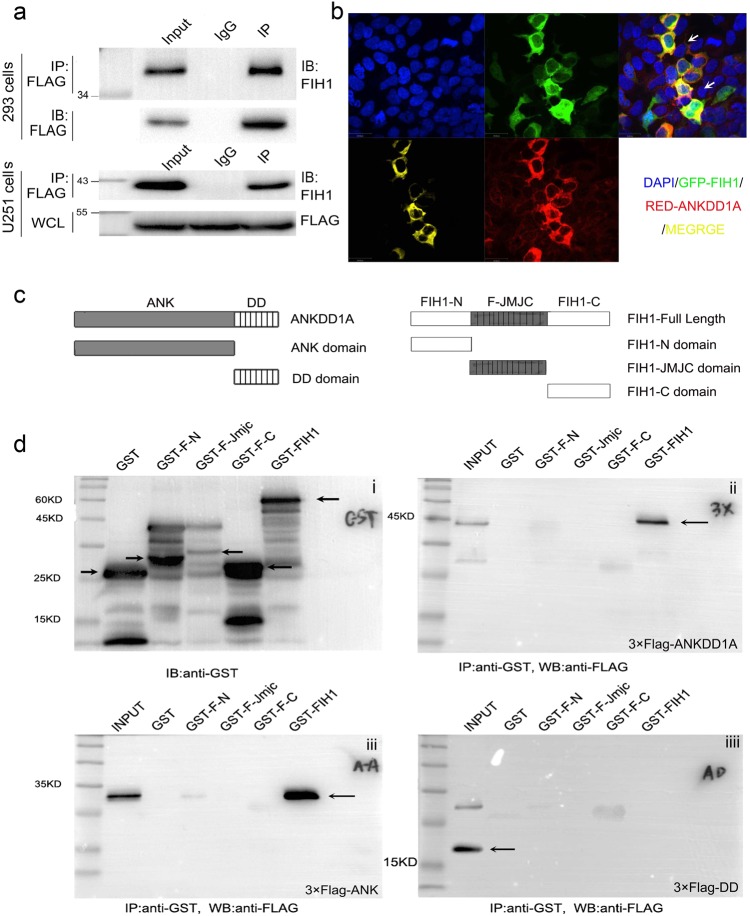
ANKDD1A interacted with FIH1. **a** HEK293(up)/U251(down) cells were transfected with Flag-ANKDD1A. Co-immunoprecipitation showed the interaction between ANKDD1A and endogenous FIH1 in HEK293/U251 cells. **b** The confocal fluorescence of HEK293 cells co-transfected with pDsRed-ANKDD1A and pEGFP-FIH1. The merged image shows the co-localization of ANKDD1A and FIH1 in the cytoplasm. **c** Schematic diagrams of ANKDD1A (left)/FIH1(right) and truncated domains. Full-length ANKDD1A, domain-Ank, and domain-DD were generated as Flag-tagged fusion proteins. Full-length FIH1, F-N domain, JMJC domain, and F-C domain were generated as GST-fusion proteins expressed in prokaryotic bacteria. **d** GST pull-down assays showed that the ankyrin domain of ANKDD1A pulled down FIH1, and the F-N domain was necessary for the interaction of FIH1 with ANKDD1A. **i** Upper left: BL21 (DE3) bacteria expressed GST. GST-fusion full-length FIH1 or different domains were precipitated by glutathione–sepharose 4B beads. Immunoprecipitated proteins were analyzed by western blot with anti-GST antibody. Arrow indicated each fusion protein. **ii** Upper right: p3 × Flag-cmv-10-ANKDD1A plasmid was transfected into HEK293 cells. After 48 h, cell lysate was incubated with GST-FIH1 full-length protein or different GST-FIH1 domains. Arrow indicated Flag-ANKDD1A protein. **iii** Bottom left: p3 × Flag-cmv-10-Ank-domain of ANKDD1A plasmid was transfected into HEK293 cells. After 48 h, cell lysate was incubated with each GST-FIH1 full-length protein or different GST-FIH1 domain for GST pulldown. Arrow indicated Flag-Ank domain protein. **iiii** Bottom right: p3 × Flag-cmv-10-DD-domain plasmid was transfected into HEK293 cells. After 48 h, cell lysate was incubated with each GST-FIH1 full-length protein or different GST-FIH1 domains for GST pulldown. Arrow indicated Flag-DD domain protein

To gain new insights into the function of ANKDD1A, the “BioGRID” (version 3.4) software was utilized to screen for potential proteins interacting with ANKDD1A. When the “high stringency” criteria were used, FIH1 and RPGRIP1L (BioGRID screen result was shown in the supplementary Fig. S1) were found to be binding partners of ANKDD1A, and FIH1 was selected for further research. To test whether ANKDD1A actually interacted with endogenous FIH1, a Flag-tagged ANKDD1A protein expression vector was transfected into HEK293 cells. The endogenous FIH1 was co-immunoprecipitated with ANKDD1A from the cell extract (Fig. 3a). We also co-expressed green fluorescent protein (GFP)-FIH1 with red fluorescent protein (RFP)-ANK in HEK293 cells and analyzed their co-localization by confocal fluorescence microscopy (Fig. 3b). The confocal results showed that ANKDD1A and FIH1 co-localized in the cellular cytoplasm of HEK293 cells. Together, these results confirmed that ANKDD1A directly binds to FIH1 in cells.

To identify the interacting regions, we subsequently performed a glutathione-S-transferase (GST) pull-down assay with ANKDD1A or different domains of ANKDD1A (Ank: ankyrin repeats domain, DD: death domain) and GST-fused FIH1, either full-length or different domains of FIH1 (F-N: N-terminal of FIH1, F-JMJC: middle domain of FIH1, or F-C: C-terminal of FIH1), as shown in Fig. 3c, d. The results revealed that full-length ANKDD1A was pulled down by either the GST-fused full-length FIH1 (Fig. 3d(ii), line 7, arrow shown) or GST-fused N-terminal domain of FIH1 (Fig. 3d ii, line 4), whereas the GST-fused F-JMJC (142-312) or F-C (313-349) was not able to pull down ANKDD1A (Fig. 3d(ii), line 5 and 6). These results indicate that the N-terminal domain of FIH1 is responsible for the interaction with ANKDD1A. In addition, Fig. 3d(iii) and (iiii) showed that the Ankyrin domain (residues 1–262) of ANKDD1A, but not the death domain (residues 263–399), precipitated with both the GST-fused full-length FIH1 (Fig. 3d(iii), line 7, arrow shown) and the GST-fused N-terminal domain of FIH1 (Fig. 3d(iii), line 4, arrow shown). Collectively, these data demonstrated that ANKDD1A binds to FIH1 directly, through the ankyrin domain of ANKDD1A and the N-terminal domain of FIH1.

### ANKDD1A suppressed the expression of hypoxic response genes by inhibiting HIF1α transcriptional activity in the hypoxia microenvironment

**Fig. 4:**
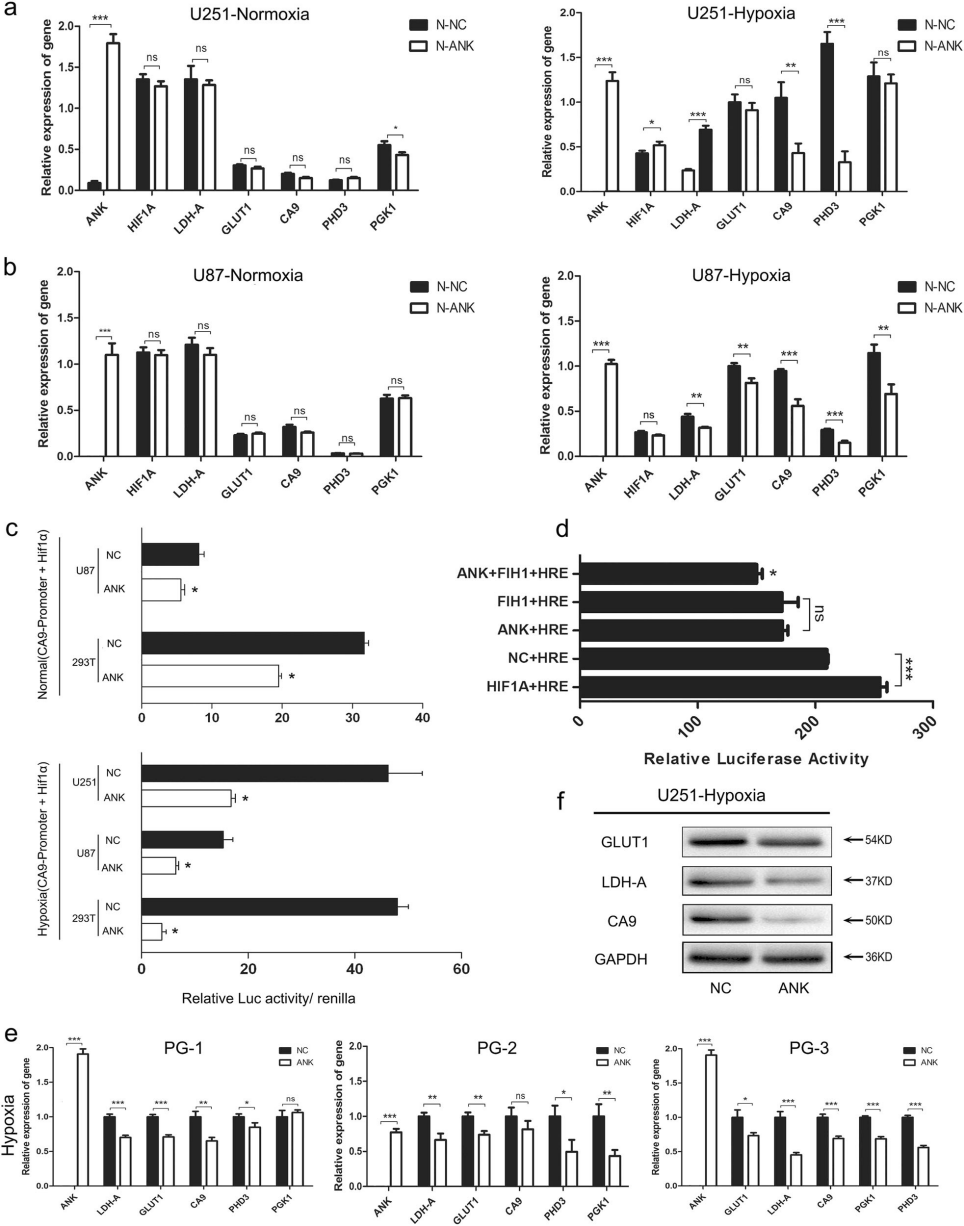
ANKDD1A reduced HIF1α transcriptional activity under hypoxia. **a**–**b** The overexpression of ANKDD1A decreased the expression of hypoxia response genes (GLUT1, LDH-A, CA9, PHD3, and PGK1) under hypoxia in U251/U87 cells. *P < 0.05, **P < 0.01, ***P < 0.001. **c** CA9 promoter was cloned into the pGL3-enhancer luciferase reporter. Luciferase reporter assays were performed 48 h after transfection with the indicated pGL3-REPORT plasmids and a Renilla transfection control plasmid that was co-transfected with ANKDD1A or a relevant scrambled control under normoxia or hypoxia. The expression of ANKDD1A dramatically decreased FLuc activity under hypoxic stress but only slightly decreased it under normoxia. *P < 0.05. **d** The HRE-luciferase reporter was used to confirm the inhibition of ANKDD1A in regulating HIF1α transcriptional activity. Luciferase activity was the lowest when co-overexpression of ANKDD1A and FIH1 was achieved. **P < 0.01, ***P < 0.001. **e** Hypoxia response gene expression was detected in astrocytoma patient-derived primary cells. The overexpression of ANKDD1A decreased mRNA expression levels of HIF1α target genes under hypoxia. *P < 0.05, **P < 0.01, ***P < 0.001. **f** Western blot detected the expression of three HIF1α transcriptional activity-dependent genes after the overexpression of ANKDD1A in U251 cell under hypoxia conditions. The overexpression of ANKDD1A also inhibited the protein expression of HIF1α target genes under hypoxia

The transcriptional activity of HIF1α is tightly regulated by FIH1, which hydroxylates the Asn803 residue of the HIF1α C-TAD (C-terminal transcription activation domain) and thereby blocks the interaction of HIF1α with the p300/CBP co-activator. Hence, we speculated that ANKDDA might participate in the HIF1α signaling pathway by interacting with FIH1. First, we investigated whether ANKDD1A affected hypoxic response gene expression and found that under low oxygen conditions (1% oxygen concentration), ANKDD1A reduced the mRNA expression level of HIF1α responsive genes such as Glut1, BNIP3, PHD3, LDH-A, CA9, and PGK1 in GBM U251 and U87 cells (Fig. 4a, b). Subsequently, we constructed a pGL3-CA9 firefly luciferase (FLuc) reporter vector driven by the promoter region of CA9, which contains hypoxia response elements (HRE). When both pcDNA3.1-ANKDD1A and pGL3-CA9 plasmids were co-transfected in HEK293T and astrocytoma cells, we found that the ectopic expression of ANKDD1A dramatically reduced the FLuc activity under hypoxic stress but only slightly decreased it under normoxia (Fig. 4c). We also used a classic HRE-luciferase vector to monitor the transcriptional activity of HIF1α when pcDNA3.1-ANKDD1A or pCDNA3.1-FIH1 was transfected in glioma cells under hypoxia. The results showed that both ANKDD1A and FIH1 directly reduced HIF1α transcriptional activity. Moreover, the relative luciferase activity was the lowest when ANKDD1A was co-expressed with FIH1 (Fig. 4d), suggesting that ANKDD1A suppressed HIF1α transcriptional activity by interacting with FIH1.

In line with this result, ANKDD1A also markedly decreased the mRNA expression levels of HIF1α target genes when ANKDD1A was transfected in three types of primary glioma cells (Fig. 4e). The overexpression of ANKDD1A also inhibited the protein expression of HIF1α target genes under hypoxia, such as LDH-A, Glut1, CA9, PHD3, and PGK1 (Fig. 4f). Taken together, ANKDD1A decreased the expression of hypoxia response genes by reducing transcriptional activity of HIF1α at low oxygen conditions.

### ANKDD1A increased HIF1α ubiquitinated degradation through upregulating FIH1 and PHD2 in the hypoxia microenvironment

**Fig. 5:**
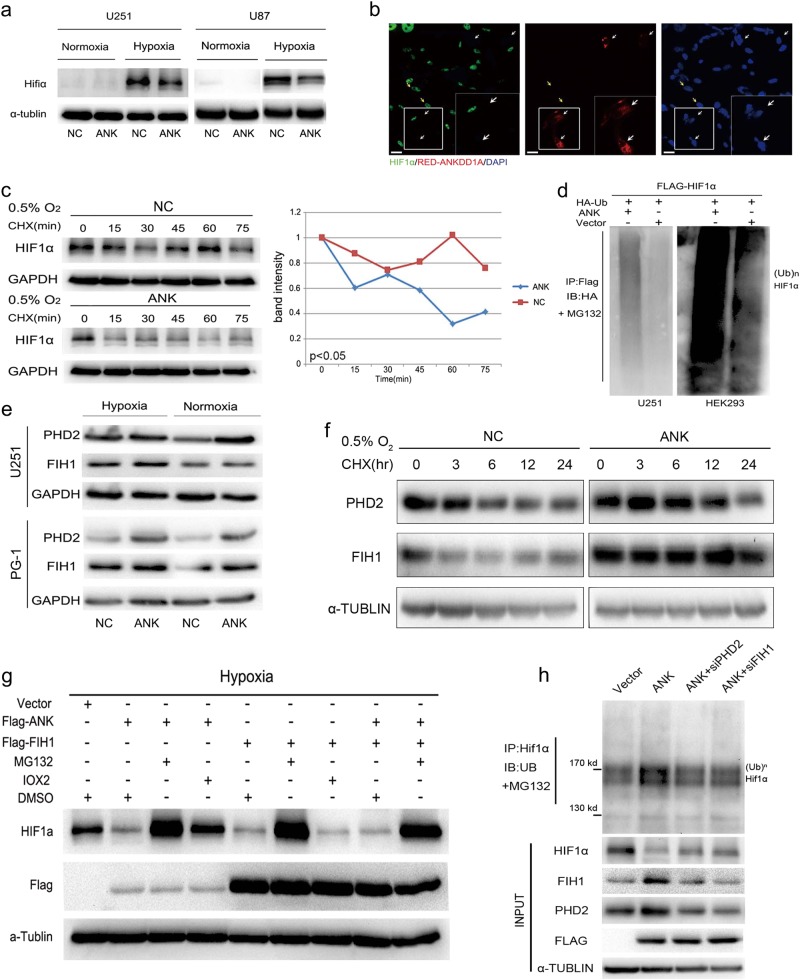
ANKDD1A reduced HIF1α stability by upregulating FIH1 and PHD2. **a** Western blot analysis for HIF1α upon the overexpression of ANKDD1A in U251 or U87 glioma cells. ANKDD1A significantly decreased HIF1α under hypoxia but had no effect on HIF1α under normoxia. **b** U251 cells were transfected with red fluorescent protein (RFP)-ANKDD1A under hypoxic conditions. Cells were stained with anti-HIF1α antibody (green) as indicated. The nuclei were stained with DAPI (blue). Scale bar, 20 μm. **c** Western blot analysis for measurement of the half-life of HIF1α after treatment with cycloheximide in U251 cells with or without ANKDD1A transfection, ANKDD1A shortened the half-life of HIF1α. **d** HIF-1α ubiquitination was assessed by anti-Flag antibody in the presence of MG132 when Flag-HIF1α, HA-ubiquitin, and pCDNA-ANKDD1A or Vector were co-transfected into U251/293 cells. ANKDD1A increased HIF1α ubiquitination. **e** Western blot analysis for PHD2 and FIH1 when ANKDD1A was transfected in U251 cells or PG-1 patient-derived primary cells. ANKDD1A increased PHD2 and FIH1 expression. **f** Western blot analysis for measurement of the half-life of PHD2 and FIH1 after treatment with cycloheximide in U251 cells with or without ANKDD1A transfection. ANKDD1A prolonged the half-life of PHD2 or FIH1. **g** Both the overexpression of ANKDD1A and FIH1 decreased the expression of HIF1α. MG132 or IOX2 restored the expression of HIF1α when ANKDD1A was overexpressed, but only MG132 treatment restored HIF1α expression when cells were transfected with FIH1. **h** HIF-1α ubiquitination was assessed by an anti-HIF-1α antibody. The ubiquitinated modification of HIF1α was decreased when silencing PHD2 or FIH1 in U251 cells stably expressing ANKDD1A

Next, we examined the effect of ANKDD1A on HIF1α expression in GBM cells. Under normoxia, we barely detected the expression of HIF1α. However, under hypoxia, HIF1α expression was markedly decreased in U251 and U87 cells transfected with ANKDD1A (Fig. 5a and supplementary Fig. S2a). We also used confocal fluorescence to examine the potential effects of ANKDD1A on HIF1α localization or expression and revealed that HIF1α remained exclusively in the nucleus under hypoxia conditions, but when in the presence of ANKDD1A expression, the HIF1α staining signal disappeared (Fig. 5b). These data indicated that ANKDD1A regulated HIF1α protein levels under hypoxia conditions.

Then, we wanted to know whether ANKDD1A altered the stability of HIF1α protein. By examining the half-life of HIF1α protein with cycloheximide treatment, a protein synthesis inhibitor, we observed that ANKDD1A shortened the half-life of HIF1α under hypoxia (Fig. 5c), suggesting that ANKDD1A decreased the protein levels of HIF1α through increasing its instability. We also examined the effect of overexpressing ANKDD1A on HIF1α ubiquitinated modification, since HIF1α is ubiquitinated by pVHL E3 ligase and rapidly degraded by the proteasome under normoxia [24]. The ubiquitinated modification of HIF1α was also increased by transfected ANKDD1A (Fig. 5d).

The stability of HIF1α is known to be regulated by PHD2, a proline-hydroxylase, and the dysregulation of FIH1 also accounts for the decreased half-life of HIF1α [25]. Consistent with this, we found that ANKDD1A expression increased FIH1 and PHD2 expression in GBM cells under hypoxia and normoxia (Fig. 5e and supplementary Fig. S2b). To test the mechanism of PHD2 and FIH1 upregulation, U251 cells transduced with ANKDD1A or vector were treated with CHX, and the half-life of PHD2 and FIH1 was analyzed. The results showed that the protein half-lives of PHD2 and FIH1 were increased in ANKDD1A-expressing U251 cells compared to vector-U251 cells (Fig. 5f). Treating GBM cells under hypoxia with MG132 (proteasome inhibitor) or IOX2 (PHD2-specific inhibitor), we found that MG132 or IOX2 increased the HIF1α protein level but had no noticeable effect on FIH1 (shown in Supplementary Fig. S3), suggesting that HIF1α was degraded in the PHD2-dependent ubiquitin–proteasome degradation pathway. In addition, we unexpectedly found that the expression of HIF1α was significantly decreased by FIH1 in GBM cells under hypoxia conditions (Fig. 5g). Although both MG132 and IOX2 restored the expression of HIF1α with overexpression of ANKDD1A, only MG132 treatment, and not IOX2 treatment, restored HIF1α expression when FIH1 was overexpressed, indicating that FIH1 mediated the degradation of HIF1α in a PHD2-independent, ubiquitin–proteasome degradation pathway. Finally, we found that the ubiquitinated modification of HIF1α was decreased when silencing PHD2 or FIH1 in U251 cells that stably expressed ANKDD1A (Fig. 5h). Taken together, these data indicated that ANKDD1A shortened the half-life of HIF1α through increasing FIH1 and PHD2 expression in GBM cells under hypoxia conditions.

### ANKDD1A disturbed the tolerance of GBM cells to hypoxia by inhibiting cell metabolism and autophagy

**Fig. 6:**
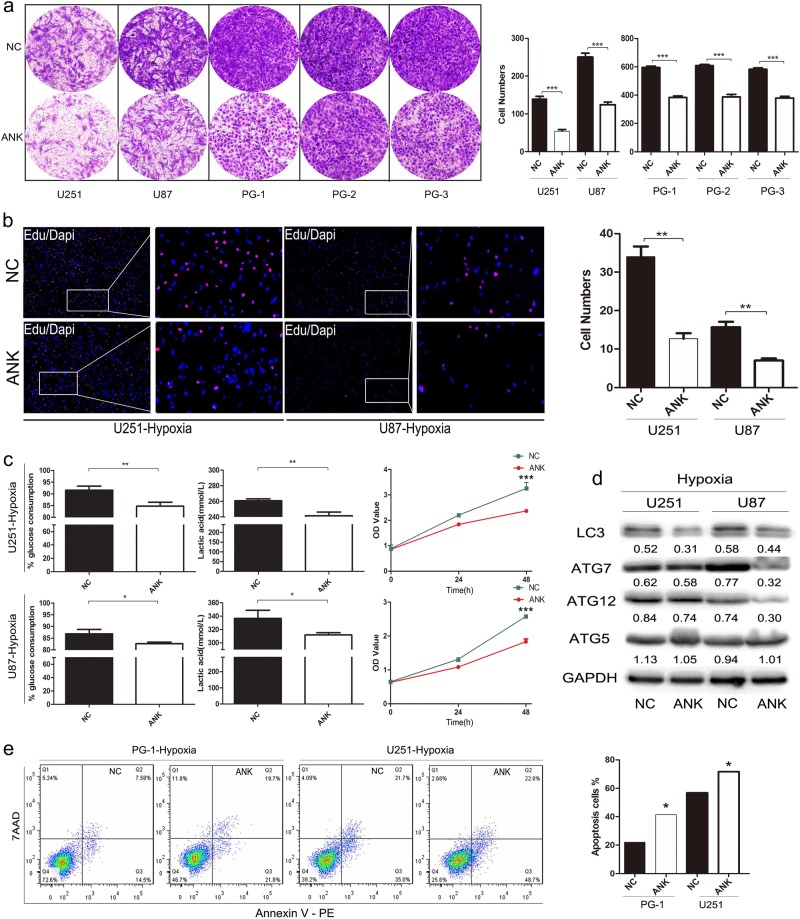
ANKDD1A significantly impaired GBM cell adaptation to hypoxia environments. **a** The inhibition of invasion by ANKDD1A in GBM cells under low oxygen culture conditions. ANKDD1A significantly decreased GBM cell invasion in hypoxia. ***P < 0.001. **b** EDU staining showed that ANKDD1A inhibited DNA replication of GBM cells. The picture on the right is the enlargement of white box of the left pictures. **P < 0.01. **c** ANKDD1A decreased cellular glucose consumption, lactic acid production, and cell proliferation under hypoxia. *P < 0.05, **P < 0.01. **d** Expression of the autophagy-related proteins ATG5, ATG7, ATG12, and LC3 was downregulated by ANKDD1A in U251/U87 cells under hypoxia. **e** Apoptosis was detected by flow cytometry. ANKDD1A increased GBM cell apoptosis under hypoxia. *P < 0.05

Hypoxia is an almost universal hallmark of solid tumors, including glioma, and the HIF1α signal pathway plays an important role in cell response to the hypoxic environment [26]. HIF1α activation would allow for the adaptation of glioma cells to hypoxia and survival. We next examined the effects of ANKDD1A on GBM hypoxic tolerance. Of note, the re-expression of ANKDD1A significantly decreased tumor cell invasion under normoxia (Fig. 2c) and hypoxia conditions (Fig. 6a). In agreement with this, EDU staining assay confirmed the inhibition of cellular DNA replication by ANKDD1A in normoxia (Fig. 2d) and hypoxia environments (Fig. 6b). Importantly, we found that the suppression function of ANKDD1A was stronger under hypoxia than normoxia in GBM cells, for the significant *p* value is much smaller in hypoxic conditions compared to the normoxic conditions.

Metabolic behaviors were changed in the hypoxia microenvironment, such as increased glycolysis and lactate metabolism, to prompt cells to adapt to hypoxic stress [27]. Thus, we next examined whether ANKDD1A causes impairment of glucose metabolism in GBM cells under hypoxia. As expected, GBM cells transfected with ANKDD1A had lower glucose uptake rates and lower levels of LDH activity compared to control (NC) under hypoxia (Fig. 6c). At the same time, ANKDD1A overexpression inhibited the proliferation (Fig. 6b) and autophagy (Fig. 6d) of both U251 and U87 cells in hypoxia conditions. Autophagy may be a self-rescue of tumor cells in hypoxia, and inhibition of autophagy may cause tumor cell death [28]. Consistently, the overexpression of ANKDD1A increased cell apoptosis under hypoxia in astrocytoma. The above data support the hypothesis that ANKDD1A significantly disturbs the tolerance of GBM cells to hypoxia, decreases cellular glucose and lactate metabolism, and induces cell apoptosis.

### ANKDD1A expression suppressed tumor growth and improved survival

**Fig. 7:**
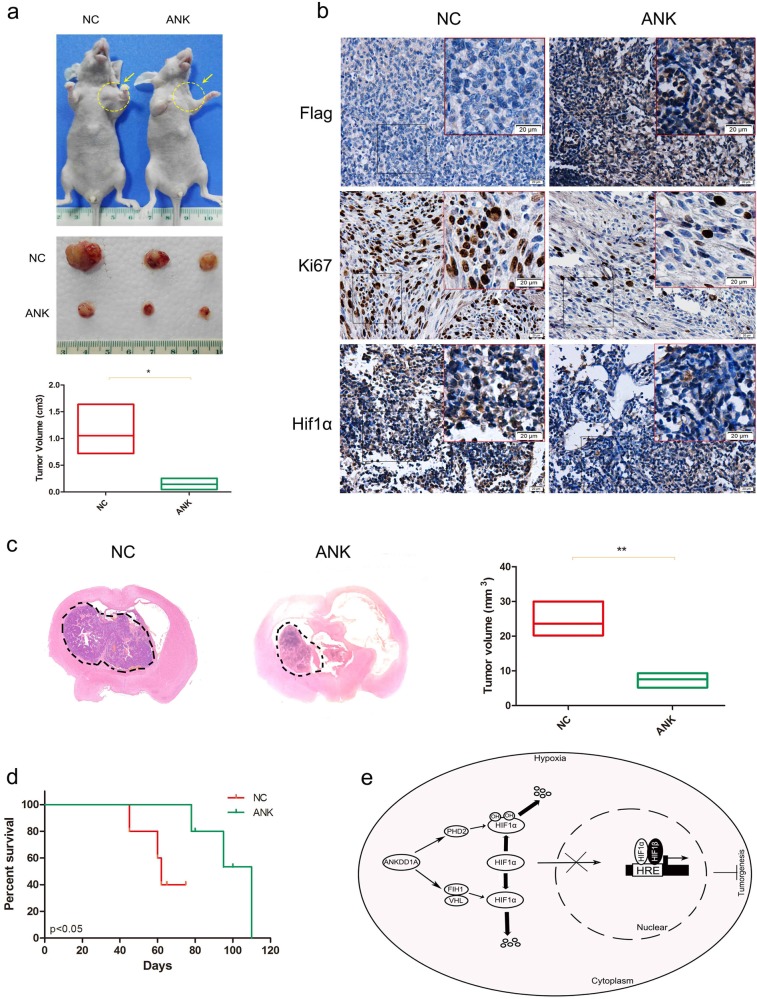
ANKDD1A expression suppressed tumor growth and improved survival. **a** The subcutaneous transplantation tumor model in nude BALB/c mice was established using U251 cells that were stably transduced with lenti-NC or lenti-ANK. The tumor size was measured. *P < 0.05. **b** IHC analysis of tumor sections showed the expression of Flag-ANK, Ki67, and HIF1α. **c** Representative images for mice brains that received injection of U251 cells overexpressing ANKDD1A (ANK) versus control (NC). **P < 0.01. **d** Kaplan–Meier survival curves of mice that were implanted with U251 cells overexpressing ANKDD1A (ANK) versus control (NC). *P < 0.05. **e** Model of the ANKDD1A-mediated pathway in regulating HIF1α stability in GBM cells under hypoxia conditions

To evaluate if ANKDD1A possessed in vivo anti-tumor capabilities, we established a subcutaneous transplantation tumor model in nude BALB/c mice using U251 cells. Nude mice were inoculated with U251 cells with a lentivirus-encoded ANKDD1A or control in the armpit of left forelimb. After 45 days, tumor volume was measured in each group. As shown in Fig. 7, the tumor volumes were smaller in mice engrafted with lenti-ANK U251 cells than in animals implanted with lenti-NC U251 cells (Fig. 7a). Moreover, the immunohistochemical staining revealed that lenti-NC tumors showed increased expression of a proliferation marker (Ki67) and HIF1α staining compared with lenti-ANK tumors (Fig. 7b). To assess whether ANKDD1A expression affected intracranial tumor growth, stereotactic intracranial injection of lenti-ANK U251 cells or lenti-NC U251 cells in immunocompromised mice was conducted. Lenti-NC tumors were strikingly more invasive and larger, generating bigger vessels in the peritumoral region that were evident on histological examination (Fig. 7c). Moreover, survival analysis showed mice intracranially engrafted with lenti-ANK U251 cells survived (*p* < 0.05) longer than animals implanted with lenti-NC U251 cells (Fig. 7d). Our studies indicated that ANKDD1A expression inhibited tumor growth in vivo and extended mouse overall survival.

## Discussion

In previous studies, we found a new hypermethylated gene in glioma, ANKDD1A, which is an unnoticed gene with unknown function. In this study, we first verified that ANKDD1A is frequently silenced or has lower expression level in glioma mainly due to its abnormal promoter CpG methylated modification. Ectopic expression of ANKDD1A inhibits the proliferation and invasion of GBM cells. Although we have investigated the mechanism of ANKDD1A standard glioma cell lines, because of some important limitations of cell lines and primary cell cultures were well acknowledged to reflect the tumor biology of glioblastoma patients. We also separated patient-derived primary cell for experiments in our study. The re-expression of ANKDD1A also inhibits the proliferation and invasion of patient-derived primary cells under normoxia and hypoxia microenvironments. Therefore, ANKDD1A could act as a tumor suppressor gene in GBM, especially under hypoxia conditions.

ANKDD1A is located at 15q22.31 and contains nine ankyrin repeats and one death domain. The ankyrin repeat is one of the most common protein–protein interaction motifs in nature. Ankyrin repeats are tandemly repeated modules of ~33 amino acids. The repeat has been found in proteins of diverse functions, including transcriptional activators, transporters, inflammatory responses, and signal transducers [18–21]. The death domain (DD) is a homotypic protein interaction module composed of a bundle of six alpha helices. DD is related in sequence and structure to the death effector domain (DED) and the caspase recruitment domain (CARD), which work in similar pathways and show similar interaction properties. Some DD-containing proteins are involved in the regulation of apoptosis and inflammation through their activation of caspases and NF-kappaB, which typically interacts with TNF (tumor necrosis factor) cytokine receptors [29, 30]. We first found and authenticated that FIH1 directly binds ANKDD1A. FIH1 is an oxygen-dependent asparaginyl hydroxylase that was initially reported to regulate the activity of HIF1α [31]. In addition to the function as HIF1α inhibitor, the FIH1 gene has also been associated with hydroxylation events in a range of certain ankyrin repeat domain (ARD)-containing proteins, including members of the IκB and Notch families [32–35]. FIH1 has been described as a regulator of the Notch pathway, metabolism and apoptosis [36, 37]. In this study, we confirmed that FIH1 interacts with ANKDD1A, and the ankyrin repeat domain of ANKDD1A directly binds to the N-terminal domain of FIH1. FIH1 catalyzes asparagine hydroxylation of HIF1α, which blocks the association of HIF1α with CBP/p300 transcriptional coactivators, thereby reducing the transcription activity of HIF1α. We propose that ANKDD1A regulates the activity of HIF1α. Supporting this hypothesis, our findings demonstrated that ANKDD1A significantly decreased transcriptional activity of HIF1α in GBM cells under hypoxia. Moreover, the relative luciferase activity controlled by the transcriptional activity of HIF1α was the lowest upon co-overexpression of ANKDD1A and FIH1, suggesting that ANKDD1A suppressed HIF1α transcriptional activity by interacting with FIH1. Since tumor cells grow rapidly and need to consume much oxygen for cellular respiration, insufficient blood vessel growth rate leads to hypoxic regions in tumors, and hypoxia is considered a harsh condition for tumor cells. Under the stressful conditions, HIF1 activation would allow tumor cells to adapt to hypoxia and survive [38]. It has been demonstrated that HIF1α is overexpressed in a broad spectrum of human malignancies [39]. Furthermore, HIF1α accumulation has been associated with poor patient survival [40]. The overexpression of HIF1α was also well described in glioma [41]. HIF1α activates target gene expression involved in vascularization, glucose transport, energy metabolism, and growth and metastasis, including VEGF, PDK1, Cyclin-D2, and CA9, and makes glioma cells adapt to hypoxic environments. Blocking HIF1α accumulation may be a therapeutic strategy for tumors. Generally, HIF1α is hydroxylated on specific proline residues by prolyl hydroxylases (PHD) and becomes a substrate for protein von Hippel–Lindau (pVHL) E3 ligase for proteasome degradation under normoxic conditions [42]. However, not only hydroxylation but also other posttranslational modifications, including SUMOylation, acetylation, methylation and phosphorylation [43–46], are known to regulate HIF1α stability. In our research, we first found that ANKDD1A plays an important role in regulating the stability of HIF1α in astrocytoma cells under hypoxic conditions. The overexpression of ANKDD1A also decreased HIF1α protein level, while treatment with MG132 blocked this effect, suggesting that ANKDD1A mediates the degradation of HIF1α in an ubiquitin-dependent manner. Of note, the FIH1 protein level was increased in GBM cells when ANKDD1A was expressed. Therefore, we proposed that the increase of FIH1 makes HIF1α more susceptible to degradation via non-PHD-mediated degradation. We provided evidence that FIH1 leads to the HIF1α destabilization under hypoxic conditions, but this effect was not blocked by IOX2, which is a direct PHD enzyme inhibitor. The most likely explanation is that FIH1 directly interacts with VHL [47]. The accumulation of FIH1 facilitates the binding between FIH1 and HIF1α, thus resulting in the degradation of HIF1α. Taken together, our findings demonstrated that ANKDD1A degraded HIF1α protein through interacting with FIH1 in GBM cells.

Extensively studied as part of HIF1 signaling, tumor cell adaptation to hypoxia benefited from metabolic reprogramming [48]. HIF1α functions as a master regulator for cellular and homeostatic response to hypoxia by activating transcription of many genes that facilitate metabolic adaptation to hypoxia, such as GLUT1, PDK1, and LDH-A. Under hypoxic conditions, glycolytic rates are enhanced, resulting in an increase in lactate production by cells expressing HIF1α. Autophagy is a cellular biological process that degrades unnecessary or dysfunctional proteins and organelles to maintain nutrient and energy homeostasis during stress conditions [49]. It has been reported that hypoxia induces autophagy in an HIF1-dependent manner [50, 51]. Our data also indicated that ANKDD1A reduced tumor cell glucose uptake and lactic acid production under hypoxia, inhibited cell autophagy, induced apoptosis, and increased the glioma patient survival.

In hypoxia, HIF1α stable existence and activation allow tumor cells to adapt to hypoxia and survival. As HIF1α is degraded and made non-functional by certain degradation mechanism under normoxia, ANKDD1A cannot function through HIF1α; therefore, it must have another function pathway under normoxia. Although we did not mention the mechanism for the biological effects of ANKDD1A in normoxia, it has been reported that FIH1 expression decreased the oncogenic potential of HNSCC cells in nomorxia [52]. Since FIH1 is located at chromosome 10q24, which is often deleted in some cancers [41], FIH1 might can also act as a tumor suppressor gene. Moreover, another article proposed PHD2 as a potential tumor suppressor in breast cancer [53]. Our results indicated that the overexpression of ANKDD1A upregulated FIH1 and PHD2 expression in both normoxia and hypoxia. We suspect that the upregulation of FIH1 and PHD2 will act as a tumor suppressor in GBM under normoxia; this may be the mechanism for ANKDD1A biological effects in normoxia.

In conclusion, this study demonstrates that ANKDD1A is a functional tumor suppressor gene, especially under a hypoxia microenvironment. ANKDD1A directly interacts with FIH1 and inhibits both the transcriptional activity of HIF1α and HIF1α-dependent gene expression by upregulating FIH1. In addition, ANKDD1A also decreased the half-life of HIF1α by upregulating FIH1, decreased glucose uptake and lactate production, inhibited glioma cell autophagy, and induced apoptosis in GBM cells under hypoxia. Moreover, ANKDD1A is highly frequently methylated in glioma, and the tumor-specific methylation of ANKDD1A indicated that it could be used as a potential epigenetic biomarker and possible therapeutic target.

## Materials and methods

### Tissue samples

Primary glioma samples and normal brain tissues were obtained from the Department of Neurosurgery, Xiangya Hospital, Hunan, China. This study was approved by the hospital institutional review board, and written informed consent was obtained from all patients. All the protocols were reviewed by the Joint Ethics Committee of the Central South University Health Authority and performed following national guidelines. Primary tumor and normal brain tissues were frozen in liquid nitrogen and stored until total RNAs or genomic DNA were extracted.

### Cell lines culture and reagents

Human glioblatoma cell lines U251 and Human Embryonic Kidney (HEK) 293 cells were maintained in DMEM medium with high glucose and sodium pyruvate, supplemented with 10% fetal bovine serum and antibiotics (100 units/ml penicillin and 100 mg/ml streptomycin). Human glioblatoma cell lines U87 were maintained in MEM medium supplemented with 10% fetal bovine serum. Cells were incubated at 37 °C in a humidified atmosphere of 5% CO_2_ in air. Cells were authenticated that it origins from ATCC by short-tandem repeat profiling. Antibodies against HIF1α(20960-1-AP), GST(66001-1-Ig), and HA(51064-2-AP) were purchased from ProteinTech. Antibodies against FIH1(sc-26219) were purchased from Santa Cruz Biotechnology (Santa Cruz, CA, USA). FLAG (F1804) was purchased from Sigma-Aldrich. Antibodies against CA9 (No. D120346), GLUT1 (No. D160433), LDH-A (No. D199841), and PHD2 (No. D122872) were purchased from Sangon Biotech. Antibodies against ATG5 (D5F5U), ATG7 (D12B11), ATG12 (D88H11), and LC3A/B (D3U4C) were purchased from Cell Signaling Technology.

### Primary astrocytoma cell culture

All samples collected had the informed consent of the patients, and all experiments using human tissues were approved by the Joint Ethics Committee of the Central South University Health Authority. Primary glioma samples (details in supplementary Table S1) were minced with GentleMACS Dissociator (Miltenyi Biotec, Gladbach, Germany) and digested with 0.25% trypsin at 37 °C for 30 min. Digestion was stopped by adding trypsin inhibitor, and cells were passed through a 40 μm nylon cell strainer (Corning, 352340) to obtain single-cell suspensions. Cells were cultured in DMEM/F12 containing 10% FBS. Glioma cells were tested with GFAP, nestin staining and subcutaneous implantation of nude mice.

### Methylation-specific PCR and bisulfite genomic sequencing

Genomic DNA was bisulfite treated and conversed by EZ DNA Methylation-Lightning™ Kit according to the manufacturer’s instructions. Then, the bisulfite-modified DNA was used for further experiments. For the BSP, 2.5 U of Taq mix (Promega) and 2 μl of 1 mM forward and reverse primers were used in a 50-μl total reaction volume. Subsequently, 100 ng of bisulfite-treated DNA was used as the template for PCR. The PCR cycles were as follows: 95 °C for 5 min, followed by 45 cycles at 95 °C for 0.5 min, 59.98 °C for 0.5 min and 72 °C for 1 min, followed by a final extension at 72 °C for 5 min. The PCR products were purified via gel extraction from a 1% agarose gel and ligated into the pGEM-T vector (takara) following the manufacturer’s instructions. The ligation products were used to transform competent *Escherichia coli* cells (strain JM109) using standard procedures, and blue/white screening was used to select a minimum of five bacterial transformants (clones). The ANKDD1A promoter of the positive clones was sequenced by Biosune (Changsha, China) and BGI (Guangzhou, China). The increase in the methylation for each sample was calculated as the percentage of unmethylated CpG dinucleotides from the total number of CpG dinucleotides that were analyzed. For the MSP, 2 U of Taq mix (Promega) and 0.8 μl of 1 mM forward and reverse primers were used in a 20-μl total reaction volume. Subsequently, 50 ng of bisulfite-treated DNA was used as the template for PCR. The PCR cycles were as follows: 95 °C for 5 min, followed by 40 cycles at 95 °C for 0.5 min, 51.5 °C for 0.5 min, and at 72 °C for 0.5 min, followed by a final extension at 72 °C for 5 min. The PCR products were separated on 1% agarose gels and analyzed via Sybrsafe staining.

### Plasmid vector

HIF1α**-**pcDNA3, HA-Ubiquitin, HRE-luciferase, and pEGFP-FIH1 plasmid were purchased from Addgene. ANKDD1A-pcDNA3.1 plasmid was purchased from Vigene Biosciences (Shandong, China). ANKDD1A was cloned into pDsRed-N1 plasmid. HIF1α was cloned into p3 × flag-cmv-10 plasmid. Upstream promoter regions (−2000 bp from TSS) of the CA9 gene were amplified from U251 cells gDNA by PCR, PCR fragments were digested with *Kpn*1/*Hin*dIII and linked to the pGL3-Enhancer Vector (Promega, WI, USA) to create plasmid pGL3-CA9. The sequences and the orientation of the cloned fragments were confirmed by direct DNA sequencing. Different domains of ANKDD1A (ARK domain, DD domain) were cloned into p3 × flag-cmv-10 plasmid by PCR and different domains of FIH1 (F-N, F-JMJC, F-C) were cloned into pGEX-4T-2 plasmid by PCR described above.

### RNA isolation and qRT-PCR

RNA was isolated from harvested cells with Trizol reagent according to the manufacturer’s instruction (Invitrogen, CA, USA), and then used for first-strand cDNA synthesis using RevertAid RT Reverse Transcription Kit (Thermo Fisher). Real-time PCR reactions were performed using SYBR Premix DimerEraser (Takara, Dalian, China) and the relative expression of genes was normalized using GAPDH as a housekeeping gene.

### Immunoprecipitation and immunoblotting

For immunoprecipitation, cells were harvested, washed with ice-cold PBS buffer, lysed in IP buffer (25 mM Tris pH 7.5, 150 mM NaCl, 1% Triton X-100, 1 mM EDTA), followed by centrifugation for 15 min at 12,000 r.p.m. Cell lysates were incubated with indicated antibodies at 4 °C for overnight. Forty microlitres protein A/G after wash twice with IP buffer were then added to the reaction mixtures and incubated for 4 h at 4 °C. After rapid centrifugation, the resulting Sepharose pellets were washed five times with IP buffer and boiled for 10 min with addition of 2× SDS loading buffer, immunoprecipitated proteins were analyzed by SDS-PAGE. For immunoblotting, cells were harvested, washed with ice-cold PBS buffer, lysed in RIPA buffer (100 mM Tris pH 7.4, 150 mM NaCl, 5 mM EDTA, 1% Triton X-100, 1% deoxycholate acid, 0.1% SDS, 2 mM phenylmethylsulfonyl fluoride, 1 mM sodium orthovanadate, 2 mM DTT, 2 mM leupeptin, 2 mM pepstatin), and centrifuged at 12,000 rpm at 4 °C for 15 min. Protein lysates were fractionated by SDS-polyacrylamide gel electrophoresis, transferred onto PVDF membranes (Merck Millipore, Germany), and then incubated with indicated primary antibodies, washed, and probed with HRP-conjugated secondary antibodies. ECL Detection System (Merck Millipore, Germany) was used for signal detection. Quantification of western blotting results was shown in Supplementary file.

### Pull-down assay

GST-fusion proteins containing various regions of FIH1 were expressed in BL21 (DE3) bacteria with pGEX-4T-2 vector and were purified. Flag-tagged various sections of ANKDD1A were transfected into HEK293 cell and lysed in the IP buffer. Then, the lysate was incubated for 4 h with GST-tagged proteins and glutathione–Sepharose 4B beads. The beads were subsequently washed four times in the IP buffer. Precipitates were separated by SDS-PAGE and detected by western blot analysis.

### Immunofluorescence

The cultured cells were plated on coverslips and transfected with indicated plasmids. After transfection for 48 h, the cells were washed three times with ice-cold PBS, fixed in 4% paraformaldehyde (PFA) at room temperature for 30 min. Cells were then washed two times with 0.1% PBS-T. For permeabilization, cells incubated with 0.25% Triton X-100 in PBS for 15 min and washed two times with 0.1% PBS-T. Cells were incubated in blocking solutions (normal goat serum), to block nonspecific binding of the antibody for 30 min, and incubated in primary antibodies diluted in PBS. After four washes with 0.1% PBS-T, cells were incubated in secondary antibodies for 1 h. After washed three times, nuclear staining was performed with DAPI (cwbiotech, Beijing, China). Coverslips were mounted and imaged by fluorescence microscope.

### Luciferase reporter assay

U251 or HEK293 cells were seeded into a 48-well plate for luciferase assays. After overnight culture, cells were co-transfected with pGL3-CA9 and equal amounts of ANKDD1A or vector. The pRL-TK control vector was transfected as a control. After 24 h of culturing under hypoxia, luciferase assays were performed using the Dual Luciferase Reporter Assay System (Promega, WI, USA). Firefly and Renilla reniformis luciferase activities were measured 24 h later. Experiments were performed in three independent replicates.

### Xenograft tumor model

All animal experiments were approved by the Animal Care and Use Committee of Central South University. For the intracranial implantation mouse model, U251-NC and U251-ANK cells were collected, resuspended at (1 × 10^6^) cells in 4 μl of serum-free medium per animal, and then stereotactically injected into the striatum (1.0 mm anterior and 2.0 mm lateral from Bregma suture and 3.5 mm below the pial surface) of nude mice. For subcutaneous tumor formation, cells (2 × 10^6^) with 100 μl of serum-free medium were injected subcutaneously at the left flank into nude mice. A total of 24 mice were used for the intracranial xenograft tumor model, including ANK overexpression groups and control groups. After intracranial injection, two mice in each group were died, and then five mice in each group were used for survival analysis, which was calculated by the Kaplan–Meier method. Five mice in each group were killed after 45 days for IHC and HE staining. HE staining was used to show the intracranial tumor size. Tumor volumes were determined according to the following formula: A × B^2^/2, where A is the largest diameter and B is the diameter perpendicular to A.

### Statistical analysis

Exclusion and inclusion criteria for animal studies were not used in this study. The investigators were not blinded to the group assignment and the outcome assessment. Data are presented as the means ± SD from at least three separate experiments, and the data were analyzed with GraphPad Prism 5 (La Jolla, CA, USA). Differences between the variables of the two groups were examined by Student’s *t* test, and one-way ANOVA was used to evaluate the differences between the variables of multiple groups. OS curves of the xenograft tumor model mice were calculated by the Kaplan–Meier method. The results were considered significant when *P* < 0.05 was obtained.

## Electronic supplementary material

Supplementary Figures and Table

Supplementary Material and Methods
