# Supplementary material for: Hypermethylated gene ANKDD1A is a candidate tumor suppressor that interacts with FIH1 and decreases HIF1α stability to inhibit cell autophagy in the glioblastoma multiforme hypoxia microenvironment
Source: Oncogene. 2018 Aug 6;38(1):103–19. doi: 10.1038/s41388-018-0423-9 (PMC6318269; doi:10.1038/s41388-018-0423-9)
Supplement: Supplementary file 1 — Supplementary Figures and Table [file 41388_2018_423_MOESM1_ESM.pdf]

Supplementary Information (SI)

Hypermethylated gene ANKDD1A is a candidate tumor suppressor that interacts with FIH1 and decreases HIF1α stability to inhibit cell autophagy in the glioblastoma multiforme hypoxia microenvironment

Jianbo Feng et al.

Supplementary Data

Table S1. Tumor type, grade, genetic and mutational profile of patient derived cells that use in this article.

Abbreviation in manuscript	Name	Tumor Grade	Tumor Type	Olig2	GFAP	Ki67	p53	MGMT methylation	IDH1	1p/19q deleted
PG-1	G20151216	grade IV	Glioblastoma	+ +	+	30% +	Mutation	Methylated	Wild	1p36
PG-2	G20151104	grade IV	Glioblastoma	+	+	40% +	Wild	Methylated	Wild	1p36&19q13
PG-3	G20151124B	GBM	Glioblastoma multiforme	+	+	30% +	Mutation	Methylated	Wild	1p36
PG-4	G20151124C	grade II	Diffuse astrocytic glioma	+	+	2% +	Mutation	Methylated	Wild	1p36
PG-5	G20151125	GBM	Glioblastoma multiforme	+	+	10% +	Mutation	Methylated	Wild	19q13
PG-6	G20151105	grade II-III	Astrocytoma	+	+	10% +	Mutation	Methylated	Wild	1p/19q intact

Figure S1, related to Result 3

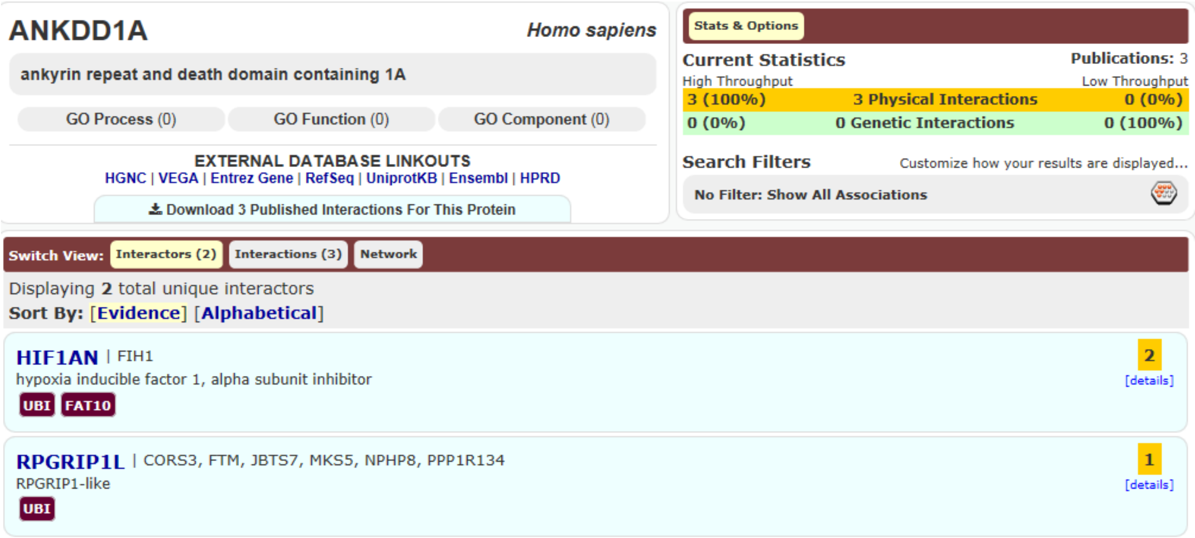

FIH1 and RPGRIP1L were predicted to interact with ANKDD1A by BioGRID screen.

Figure S2, related to Result 5

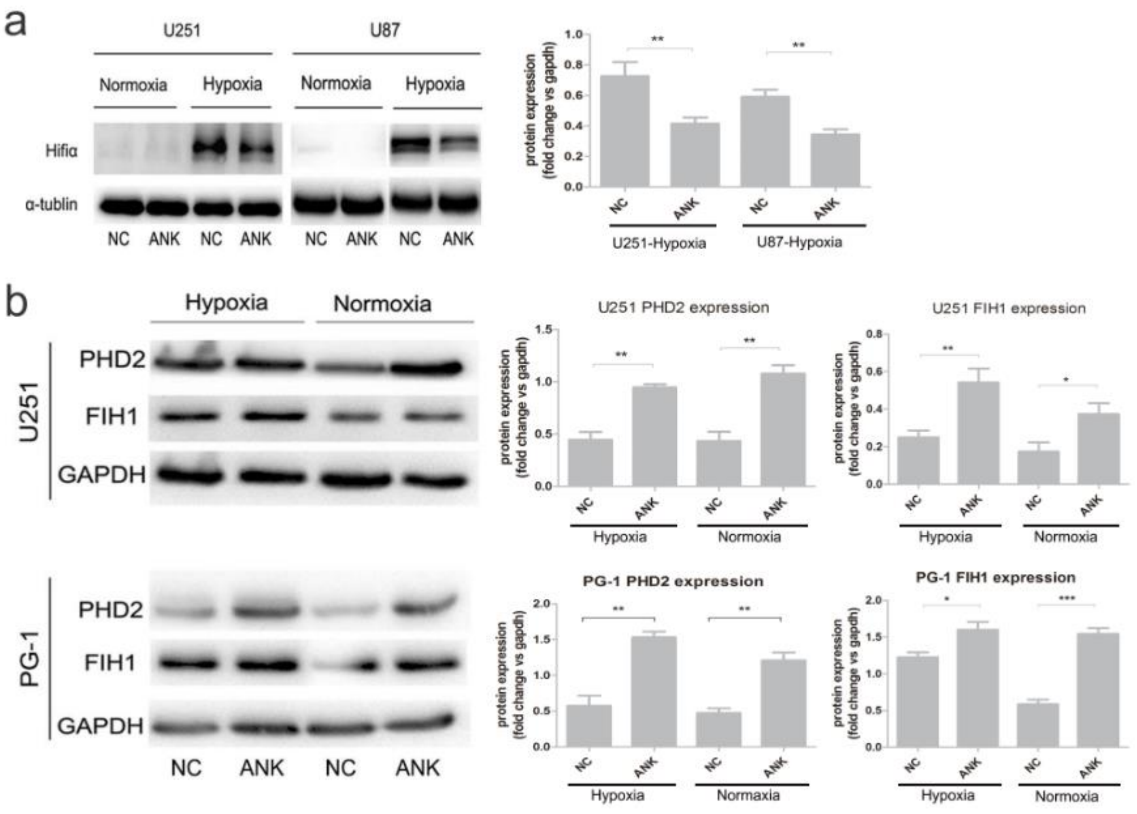

Quantification assay of western blotting of Figure 5a (**a**) and Figure 5e (**b**).

Figure S3, related to Result 5

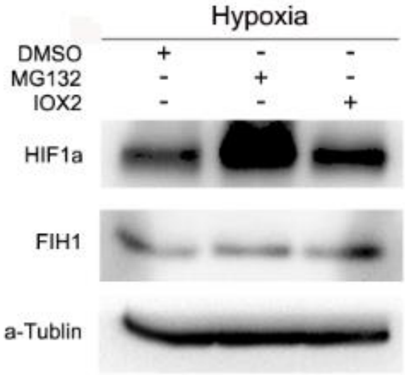

MG132 (proteasome inhibitor) or IOX2 (PHD2-specific inhibitor) increased the HIF1α protein level but had no noticeable effect on FIH1 in GBM cells under hypoxia.
