# Supplementary material for: Hypermethylated gene ANKDD1A is a candidate tumor suppressor that interacts with FIH1 and decreases HIF1α stability to inhibit cell autophagy in the glioblastoma multiforme hypoxia microenvironment
Source: Oncogene. 2018 Aug 6;38(1):103–19. doi: 10.1038/s41388-018-0423-9 (PMC6318269; doi:10.1038/s41388-018-0423-9)
Supplement: Supplementary file 2 — Supplementary Material and Methods [file 41388_2018_423_MOESM2_ESM.pdf]

Supplementary Material and Methods

In Vitro Invasion Assay

Cells invasion was measured using 24-well Fluoroblok inserts (8μm Becton Dickinson). Briefly, U251, U87, PG-1, PG-2 or PG-3 cells (50,000 cells per insert) were suspended in serum-free medium over a Matrigel coating (Becton Dickinson), and the lower chamber was cultured with 10% FBS-containing growth medium as a chemoattractant. Hydroxyurea was added into cells culture medium to inhibit cells growth which eliminates the effect of proliferation on cells invasion. Cells which invaded after 16 hr were stained in 4 mg/ml Calcein AM dye, and then counted under a light microscope.

CCK-8 cell proliferation assay

Cells proliferation was determined using a cell counting kit-8 (CCK-8) (Dojido, Kumamoto, Japan) assay according to the manufacturer's instructions. Briefly, 3,000 cells in 150 μl of medium were plated on a 96-well plate and cultured for 1-3 days. After the incubation period, 15 μl of CCK-8 was added to each well, and cells were further incubated for 3 h at 37 ℃. Absorbance was then measured at 450 nm using a microplate reader (Thermo Fisher Scientific, USA).

EDU staining assay

Cells DNA replication was determined using EdU kit (RiboBio, Guangzhou, China) according to the manufacturer's protocol. Briefly,

5,000 cells in 150 μl of medium were plated on a 96-well plate, and after cultured for 4 hr, medium was replaced by 10μM EdU medium and cultured for another 12hr. After Apollo staining and nuclear staining, cells were counted using a fluorescent microscope.

Apoptosis measurement

Apoptosis was assessed by labeling cells with annexin-V-FITC and propidium iodide (PI). Cells were then analyzed (n = 20,000) by flow cytometry (Accuri C6 cytometer, Becton-Dickinson). Annexin V-positive cells (PI negative and positive) were counted as apoptotic.

Immunohistochemistry

Mice tumor paraffin sections were dewaxed, rehydrated and antigen retrievaled. Sections were blocked with 3% hydrogen peroxide for 10 min and normal goat serum for 30 min at room temperature. Then the sections were incubated with anti-FLAG (Sigma-Aldrich), HIF1α (Proteintech) and KI67 (Bosterbio) antibodys for 12 h at 4 ℃ and incubated with biotinylated secondary antibody (Maxim Biotechnologies) for 30 min at room temperature and streptavidin conjugated HRP (Maxim Biotechnologies) for 30min. Staining was visualized with 3, 3-diaminobenzidine (DAB; Maxim Biotechnologies) and counterstained with hematoxylin.
